# Neuroinflammation in neurodegenerative disorders: the roles of microglia and astrocytes

**DOI:** 10.1186/s40035-020-00221-2

**Published:** 2020-11-26

**Authors:** Hyuk Sung Kwon, Seong-Ho Koh

**Affiliations:** 1 Department of Neurology, Hanyang University College of Medicine, Seoul, Republic of Korea; 2 Department of Translational Medicine, Hanyang University Graduate School of Biomedical Science & Engineering, Seoul, Republic of Korea

**Keywords:** Neuroinflammation, Neurodegenerative diseases, Microglia, Astrocytes

## Abstract

Neuroinflammation is associated with neurodegenerative diseases, such as Alzheimer’s disease, Parkinson’s disease, and amyotrophic lateral sclerosis. Microglia and astrocytes are key regulators of inflammatory responses in the central nervous system. The activation of microglia and astrocytes is heterogeneous and traditionally categorized as neurotoxic (M1-phenotype microglia and A1-phenotype astrocytes) or neuroprotective (M2-phenotype microglia and A2-phenotype astrocytes). However, this dichotomized classification may not reflect the various phenotypes of microglia and astrocytes. The relationship between these activated glial cells is also very complicated, and the phenotypic distribution can change, based on the progression of neurodegenerative diseases. A better understanding of the roles of microglia and astrocytes in neurodegenerative diseases is essential for developing effective therapies. In this review, we discuss the roles of inflammatory response in neurodegenerative diseases, focusing on the contributions of microglia and astrocytes and their relationship. In addition, we discuss biomarkers to measure neuroinflammation and studies on therapeutic drugs that can modulate neuroinflammation.

## Background

With the increase in life expectancy, the global socioeconomic impact of neurodegenerative diseases, including Alzheimer’s disease (AD), Parkinson’s disease (PD), and amyotrophic lateral sclerosis (ALS), is increasing considerably [1]. However, the pathological mechanisms underlying neurodegenerative diseases are not fully understood. Several factors including genetic, environmental, and endogenous factors are involved. Abnormal protein dynamics, oxidative stress with reactive oxygen species, mitochondrial dysfunction, DNA damage, dysfunction of neurotrophins, and neuroinflammatory processes are considered to be common pathophysiological mechanisms [2]. Neuroinflammation is a defense mechanism that initially protects the brain by removing or inhibiting diverse pathogens [3]. This inflammatory response can have beneficial effects by promoting tissue repair and removing cellular debris. Sustained inflammatory responses, however, are detrimental, and they inhibit regeneration [4, 5]. Inflammatory stimulation can persist due to endogenous (e.g., genetic mutation and protein aggregation) or environmental (e.g., infection, trauma, and drugs) factors [6, 7]. The persistent inflammatory responses involve microglia and astrocytes and can lead to neurodegenerative diseases [4].

Two categories of cells populate the central nervous system: neurons and glial cells [8]. Glial cells do not produce electrical impulses, and they were considered as supporting cells for neurons. It has been revealed that glial cells are superior to neurons in cellular diversity and function [9]. Glial cells, including astrocytes, oligodendrocytes, and microglia, can regulate neuronal activity [8, 10]. Microglia and astrocytes serve diverse functions including innate immune responses in the brain. Traditionally, both can be classified into two opposing phenotypes: neurotoxic and neuroprotective. Microglia are divided into the M1 (classical activation) and M2 (alternative activation) phenotypes based on their activation status [6, 11]. Similar to the microglia, astrocytes can produce pro-inflammatory or immunoregulatory mediators according to the phenotype of the polarization status [7]. However, microglia and astrocytes are considered to have multiple reactive phenotypes related to the type and stage of neurodegenerative diseases and the regional location [12–14]. Furthermore, the changes in phenotypes of microglia and astrocytes, their loss of neuroprotective functions, and their gain of neurotoxic functions are complicated and may differ with the stage and severity of neurodegenerative diseases. Therefore, the simple dichotomized classification cannot reflect the various phenotypes of microglia and astrocytes [12]. For these reasons, the use of the M1/M2 and A1/A2 nomenclature was limited in this manuscript, appearing only directly from the references where they were used. However, they should be considered as being on a spectrum, rather than being two distinct populations. This complexity could be the reason why trials of anti-inflammatory drugs have, to date, failed to show significant therapeutic effects. Here, we review the roles of inflammatory responses in neurodegenerative diseases, such as AD, PD, and ALS, focusing on the roles of microglia and astrocytes and their relationships. Recommendations for the success of clinical trials are also made. In addition, biomarkers to measure neuroinflammation and studies on drugs that can modulate neuroinflammation are also discussed.

### Microglia

Microglia are ubiquitously distributed in the brain and are the principal innate immune cells and the first responders to pathological insults [15, 16]. The proportion of microglia ranges 5–12% of the total cell population in the mouse brain depending on the location, and they have diverse morphologies: compact round, longitudinally branched, and radially branched [17]. They are involved in homeostasis and host defense mechanisms by participating in three essential functions [18]. The first function is detecting changes in their environment using their sensomes, which are encoded by various genes [19]. The second is the physiological housekeeping function, which includes migrating to injured sites, remodeling synapses, and maintaining myelin homeostasis [18, 20]. The third is protecting against injurious stimuli, including pathogen-associated molecular patterns (PAMPs) and damage-associated molecular patterns (DAMPs). Cellular receptors such as toll-like receptors (TLRs), nuclear oligomerization domain-like receptors, and viral receptors are expressed on microglia, and can recognize PAMPs and DAMPs [6, 7]. In response to such stimuli, microglia produce proinflammatory cytokines, such as tumor necrosis factor (TNF)-α, interleukin (IL)-1β, IL-16 and chemokines, including the C-C motif chemokine ligand 2 (CCL2) and IL-18, to recruit additional cells and remove pathological agents [6, 18]. However, although neuroinflammation is a neuroprotective mechanism, sustained neuroinflammation can induce neurotoxicity and is related to neurodegeneration [18]. In addition, microglia priming with aging and chronic stress shows a dystrophic morphology and an exaggerated inflammatory response [21].

Microglial activation can be assessed by imaging and fluid biomarkers. ^11^C-(R)PK11195 positron emission tomography (PET) can be used to quantify microglial activation via the binding capacity of ^11^C-(R)PK11195 to the translocator protein that is overexpressed in activated microglia [22, 23]. The soluble triggering receptor expressed on myeloid cells 2 (sTREM2), which is a cleavage product of TREM2 expressed on the cell surface of microglia [24, 25], is a fluid biomarker of microglial activation. Recent studies have shown that the cerebrospinal fluid (CSF) level of sTREM2 is correlated with plasma sTREM2 level, suggesting that the CSF sTREM2 is a potential biomarker for microglial activation [25, 26].

**Fig. 1:**
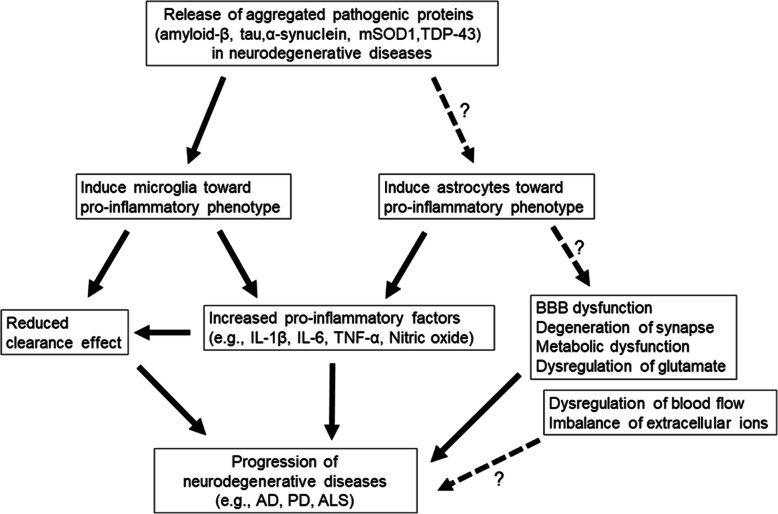
Potential relationships between neurodegenerative diseases and glial cells. The release of aggregated pathogenic proteins such as amyloid-β, tau, α-synuclein, mSOD1, and TDP-43, into the extracellular space drives the changes of microglia and astrocytes into their pro-inflammatory phenotypes. The predominance of the pro-inflammatory phenotype of microglia results in the increase of pro-inflammatory factors and a decrease of the phagocytic effect. The pro-inflammatory-phenotype astrocytes release pro-inflammatory factors, which can dysregulate the synaptic function, the blood-brain barrier, metabolic function, glutamate, extracellular ions, and blood flow. Ultimately, this can lead to neurodegenerative disease progression. A dotted line with a question mark represents a possible relationship, with a lack of evidence for a direct association

**Fig. 2:**
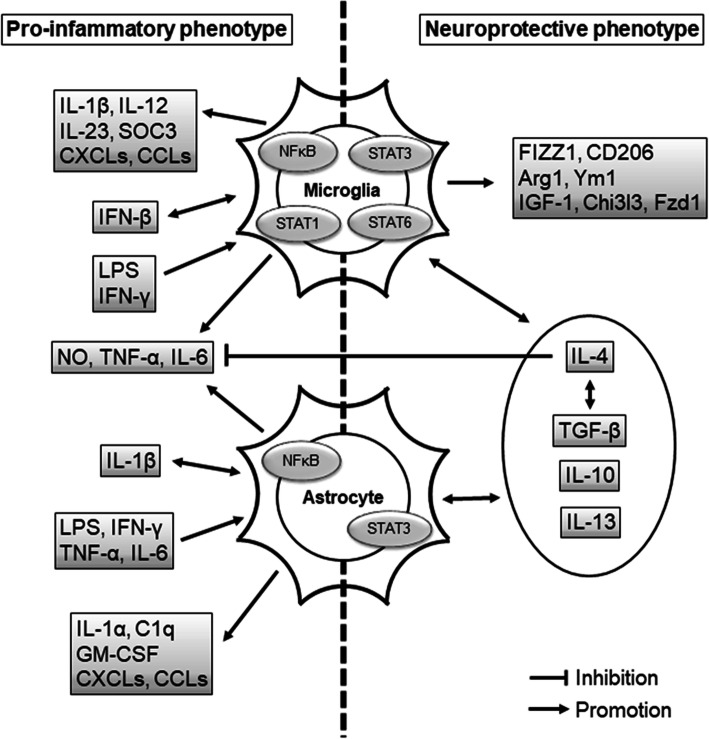
Proposed signals associated with microglia and astrocytes. The pro-inflammatory microglia are activated by IFNs and LPS via the activation of NFκB and STAT1, and then release IL-1β, IL-12, IL-23, SOC3, CXCLs, CCLs, NO, TNF-α, and IL-6. The neuroprotective microglia are promoted by IL-4, IL-13, IL-10, and TGF-β via the activation of STAT3 and STAT6. The M2 microglia enhance the neurotrophic factor (IGF-1), FIZZ1, CD206, Arg1, Ym1, Chi3l3, Fzd1, IL-13, IL-10, IL-4, and TGF-β. The activation of NFκB induces pro-inflammatory astrocytes. The pro-inflammatory astrocytes are affected by IL-1β, IFN-γ, LPS, TNF-α, and IL-6, and they produce IL-1α, C1q, GM-CSF, CXCLs, CCLS, TNF-α, IL-6, and NO. The activation of STAT3 induces neuroprotective astrocytes. The neuroprotective astrocytes interact with anti-inflammatory cytokines such as IL-13, IL-10, TGF-β, and IL-4; IL-4 and TGF-β coordinate to promote protective effects, and IL-4 suppresses TNF-α, IL-6, and NO. CCL: C-C-motif chemokine ligand; CXCL: C-X-C motif chemokine ligand; GM-CSF: granulocyte-macrophage colony-stimulating factor; IFN-γ: interferon γ; IL: interleukin; LPS: lipopolysaccharide; NFκB: nuclear factor κB; NO: nitric oxide; STAT: signal transducers and activators of transcription; TNFα: tumor necrosis factor α

**Fig. 3:**
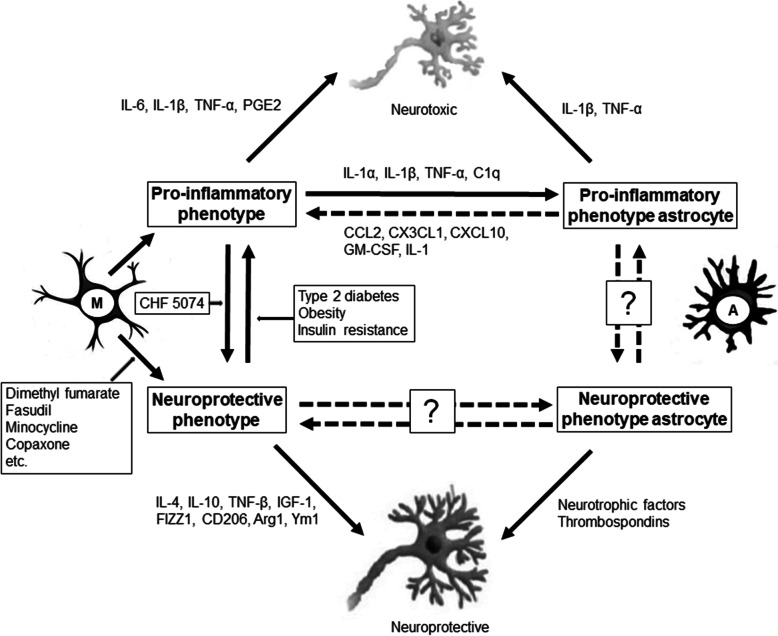
Schematic of microglial activation, astrocyte activation, and their relationship. The pro-inflammatory phenotypes are neurotoxic, while the neuroprotective phenotypes are neuroprotective. CHF 5074 polarizes microglia from the pro-inflammatory to the neuroprotective phenotype. Microglia can switch from the neuroprotective to the pro-inflammatory phenotype in the context of type 2 diabetes, obesity, and insulin resistance. Some candidates (dimethyl fumarate, fasudil, minocycline, and copaxone) can potentiate the neuroprotective polarity of microglia. The pro-inflammatory microglia secret IL-1α, IL-1β, TNF-α, and C1q, which can change astrocytes into the pro-inflammatory phenotype. The pro-inflammatory astrocytes secret CCL2, CX3CL1, CXCL10, GM-CSF, and IL-1, which in turn activate the pro-inflammatory microglia. The phenotype transition of astrocytes remains to be clarified. The dotted lines with question marks represent a possible relationship, with a lack of evidence for direct association. M: resting microglia; A: astrocytes. Other abbreviations as in Fig. 2

Microglia in the central nervous system (CNS) can be pro-inflammatory or neuroprotective, depending on their activation status. Pro-inflammatory cytokines are debris from pathogens or damaged cells, and they activate the resting microglia to express pro-inflammatory factors such as IL-1β, TNF-α, IL-6, nitric oxide (NO), and proteases, which have detrimental effects in neurodegenerative diseases (Fig. 1, Fig. 2) [6, 13]. In contrast, IL-4, IL-10, IL-13, and transforming growth factor-β (TGF-β) activate neuroprotective microglia, which leads to the release of diverse factors including FIZZ1, Chitinase-3-Like-3 (Chi3l3), Arginase 1, Ym1, CD206, insulin-like growth factor 1 (IGF-1), and Frizzled class receptor 1 (Fzd1) (Fig. 2) [6, 13, 27, 28]. These factors from microglia may be associated with neuroprotection and tissue healing (Fig. 3). For example, IL-4 is known to suppress the release of pro-inflammatory cytokines, i.e., IL-6, TNF-α, and NO [29, 30].

Switching between these two phenotypes may affect remyelination, which is associated with aging [7, 31]. Obesity, insulin resistance, and type 2 diabetes are known to impact the transition of microglia from the neuroprotective phenotype to the neurotoxic phenotype (Fig. 3) [28, 32, 33]. Several candidates and factors have been identified to potentiate the neuroprotective polarity: fasudil (Rho kinase inhibitor), Jumonji domain-containing 3 (Jmjd3, H3K27me3 demethylase), minocycline, Copaxone (glatiramer acetate), dimethyl fumarate (Tecfidera), cromolyn, CHF 5074, fingolimod, masitinib, glycogen synthase kinase-3 inhibitor, histone deacetylase inhibitor, peroxisome proliferator-activated receptor, adenosine monophosphate-activated protein kinase, and Janus kinase/signal transducers and activators of transcription (JAK/STAT) inhibitors (Fig. 3) [34–39]. The proportion of each phenotype can differ depending on the stage of neurodegenerative diseases [28]. Treatment targeting the phenotype balance may have different effects, depending on the time window [28]. Therefore, balancing and switching between the phenotypes of microglia at specific times and in specific patients may be important for modulating the progression of neurodegenerative diseases. The drugs that modulate microglial activation are more likely to exhibit protective effects in a clinical trial that 1) employ participants with more pro-inflammatory than neuroprotective microglial phenotypes, 2) enroll participants who are likely to show progression within a few years, as it is hard to follow patients for longer duration of years in clinical trials, and 3) have confirmed the pathology of the disease, such as amyloidopathy or tauopathy; without a pathological insult, the glial cells may not change. Further studies are warranted to investigate the appropriate time window and patients to demonstrate the clinical benefits of treatments targeting the microglial state.

### Astrocytes

Astrocytes are the most common glial cells in the brain [40]. Although they were initially considered to only have passive functions, recent studies have discovered that astrocytes play active and essential roles in brain homeostasis [41]. They regulate blood flow, maintain the blood-brain barrier (BBB), provide energy metabolites to neurons, modulate synaptic activity, control neurotrophin secretion, remove dead cells, as well as regulating the extracellular balance of ions, fluid and transmitters, and scar formation [40–42]. Currently, glial fibrillary acidic protein (GFAP), S100B, YKL040, and *D*-serine are assessed as CSF biomarkers and GFAP and S100B as blood biomarkers [43]. For imaging biomarkers, magnetic resonance spectroscopy, ^11^C-deuterium-*L*-deprenyl (^11^C-DED) PET, and ^11^C-BU PET are used to assess the astrocyte reactivity [43]. Changes in molecular expression and morphology of astrocytes measured by GFAP can indicate the severity of reactive astrogliosis, which is a hallmark of CNS pathology [42]. Defects of astrocytes during the early phase of injury including spinal cord injury (SCI) and experimental autoimmune encephalomyelitis (EAE), are consistently correlated with exacerbated clinical outcomes, neuroinflammation, BBB alteration, and neuronal death [40], while on the other side, a study in a chronic experimental EAE mouse model has shown that during chronic CNS inflammation, astrocytes produce lactosylceramide (LacCer), which promotes inflammation and neurodegeneration [44]. These results indicate that the effect of astrogliosis can be beneficial or detrimental, depending on the time, specific disease, and different stimuli from the microenvironment, such as microglia.

Astrocytes may have multiple simultaneous reactive profiles, but with a continuous spectrum. Therefore, the heterogeneity of reactive astrocytes should be investigated further [13]. Similar to microglia, astrocytes have pro-inflammatory and immunoregulatory (neuroprotective) subpopulations. The pro-inflammatory reactive astrocytes upregulate several genes (e.g., complement cascade genes) and induce pro-inflammatory factors (e.g., IL-1β, TNF-α, and NO), which are known to have harmful functions (Fig. 1) [6, 13]. In comparison, the neuroprotective reactive astrocytes upregulate many neurotrophic factors and thrombospondins (Fig. 3) [13]. The anti-inflammatory cytokines, such as IL-4, IL-13, and IL-10, may induce neuroprotective activation of astrocytes, and these alternatively activated astrocytes may release IL-4, IL-10, and TGF-β (Fig. 2) [41].

Inflammatory mediators secreted by pro-inflammatory microglia, such as IL-1α, IL-1β, TNF-α, and C1q, may activate pro-inflammatory astrocytes and induce a secondary inflammatory response (Fig. 3) [45, 46]. Detrimental astrocytic signaling pathways can be induced by several other cytokines, sphingolipids (sphingosine 1-phosphate and LacCer), and neurotrophins [40]. Astrocytes upregulate the transmembrane receptors for IL-17 and tropomyosin receptor kinase B (TrkB) during neuroinflammation. The binding of IL-17 to its receptors may result in the recruitment of nuclear factor κB (NFκB) activator 1 (Act1) and the production of pro-inflammatory cytokines [47]. Conditional mice lacking TrkB can be protected from EAE-induced neurodegeneration, while stimulation of TrkB by the agonist brain-derived neurotrophic factor (BDNF) has detrimental effects on neurons [48].

In contrast, astrocytes that respond to certain pathways are protective, since inhibition of the mediators of these protective pathways worsens neuroinflammation and neuronal cell death. The first protective pathway is mediated by glycoprotein gp130; it is related to the SHP2/Ras/ERK activation, and limits neuroinflammation [49]. A lack of gp130, a signal transducer for the IL-6 cytokine family, worsens the CNS injury of *Toxoplasma* encephalitis and EAE in mice [49, 50]. The second protective pathway is mediated by TGFβ, which has important immunosuppressive properties. The astrocytic TGF-β signaling may mediate the inhibition of NFκB signaling and reduce neuroinflammation after a stroke or *Toxoplasma* infection [51, 52]. The third protective pathway is mediated by interferon (IFN)-γ signaling. Although IFN-γ is a pro-inflammatory cytokine, the inhibition of its signaling in astrocytes worsens the mortality and leukocyte infiltration during the late stage of EAE in mice [53]. Finally, the estrogen receptor (ER) α signaling pathway in astrocytes has demonstrated anti-inflammatory and neuroprotective effects in various neurological disease models [54].

The transcription factor signal transducer and activator of transcription 3 (STAT3) is expressed in astrocytes and phosphorylated after injury. Ablation of STAT3 in astrocytes aggravates the infiltration of inflammatory cells, neuronal loss, and demyelination after SCI in mice [55]. BDNF secreted by activated astrocytes can enhance STAT3 activation [56]. In another in vivo and in vitro study involving an SCI mouse model, the knock-out of STAT3 attenuated astrogliosis and disrupted scar formation, which were associated with the worsening of inflammation and increased lesion volume [57]. Therefore, STAT3 seems to be critically involved in reactive astrogliosis, and is associated with a neuroprotective effect.

The JAK-STAT3 pathway may mediate the neuroprotective functions of reactive astrocytes. However, the molecular basis for the induction of the neuroprotective reactive astrocytes is unclear [58]. In addition, there could be more states of polarization than just proinflammatory or neuroprotective [13]. Therefore, the heterogeneity and molecular basis of reactive astrocytes should be investigated further.

### AD

AD is the most common form of dementia and is pathologically characterized by extracellular accumulation of amyloid-beta (Aβ)-containing plaques and development of intracellular neurofibrillary tangles composed of hyperphosphorylated tau protein [16, 59]. In addition, neuroinflammation contributes to the pathogenesis of AD [16], as inflammatory responses have been repeatedly demonstrated in AD. For example, researchers have found higher TNF-α (pro-inflammatory cytokine) and lower TNF-β (anti-inflammatory cytokine) levels in the CSF of mild cognitive impairment patients who progressed to AD, compared with the controls who did not progress to AD [60]. Some cytokines including IL-1β, IL-6, and TNF-α have slowly increased levels from the early stage of the disease, while the levels of other cytokines including IL-18, MCP-1, and IP-10 can peak at a certain stage of the disease [61]. Although current publications have inconsistent results, it has been clear that neuroinflammation occurs early in AD, and may trigger the progression of this disease.

Morphological changes of microglia and astrocytes surrounding the senile plaques are also indicative of the neuroinflammatory response [6]. Both microglia and astrocytes interact with Aβ. Dysfunctions of microglia and astrocytic metabolism can result in the accumulation of Aβ [18, 62]. Aβ in turn activates microglia and astrocytes through TLRs to release neuroinflammatory mediators that promote neurodegeneration [4, 6].

Microglia can be neuroprotective by degrading and removing Aβ and tau [63, 64]. However, the persistent interaction between Aβ and Aβ-induced pro-inflammatory cytokines overwhelms the clearance ability of microglia [18]. Increases in the size and number of Aβ plaques during the late-onset form of AD may reflect the decreased clearance ability of microglia [65]. Microglia that surround the Aβ plaques are generally of the neuroprotective phenotype, labeled as Ym1, at the beginning of Aβ pathology; but they later switch to the neurotoxic (pro-inflammatory) phenotype during the advanced stage of the disease [28, 66]. The pro-inflammatory cytokines decrease the phagocytic activity of microglia, and they are also likely to transform microglia into the pro-inflammatory phenotypes. In addition, the pro-inflammatory microglia increase the phosphorylation of tau and exacerbate tau pathology [67]. This indicates that microglia in AD are involved in the Aβ and tau pathologies. In a recent study, microglial activation, as measured by ^11^C-(R)PK11195, decreased longitudinally in patients with mild cognitive impairment and increased longitudinally in AD patients [68], suggesting that two peaks of microglial activation may be present in AD. Although the ^11^C-(R)PK11195 cannot differentiate between the phenotypes of microglial activation, the first and last peaks may be neuroprotective and pro-inflammatory, respectively, as microglia are known to change from the neuroprotective to the pro-inflammatory activation phenotypes during aging [68, 69]. In addition, the microglial activation is significantly correlated with amyloid deposition (measured by ^11^C-PIB PET) [68], but not significantly correlated with tau accumulation (measured by ^18^F-AV-1451 PET) in AD [23]. This difference can be explained by the stage of the disease and the regional location in the brain. CSF sTREM2, a marker of microglial activation, is also increased in AD patients compared with the healthy controls [25]. However, several studies on sTREM2 have contradictory findings and the discriminative power of sTREM2 for AD is low. Further studies are required to establish the utility of sTREM2 in clinical practice [70].

Pro-inflammatory reactive astrocyte phenotypes have been linked to synaptic degeneration and glutamate dysregulation [13, 41]. Knockout of astrocytic glutamate transporters EAAT1 (glutamate/aspartate transporter, GLAST) and EAAT2 (glutamate transporter-1, GLT-1) caused excitotoxicity and synaptic hyperexcitability in an AD model [71–73]. In the AD mouse model, calcineurin (Ca^2+^/calmodulin-dependent phosphatase), a nuclear factor of the activated T-4 cell signaling pathway, has been found to link between astrocyte activation and hyperexcitability during AD [72]. The astrocytes alter their function and morphology during AD, and may have different functions as AD progresses [43].

Most clinical trials for anti-inflammatory drugs in AD patients, including aspirin, prednisone, naproxen, diclofenac, indomethacin, and celecoxib, have failed to show definite improvements, even revealing some detrimental effects [74–79], although some have shown modest beneficial results. A subgroup analysis showed that mild-to-moderate AD patients who were *APOE* ε4 carriers benefitted from ibuprofen over the 12-month trial duration [80]. Another study revealed protective effects of indomethacin in mild-to-moderate AD patients over 6 months, but the patient dropout rate was high, which weakened the significance of the study [81]. The failure of the clinical trials may be explained by the possible impact of the anti-inflammatory drugs on the protective effects of glial cells, the selection of participants, and the short-term follow-up period. For example, the Alzheimer’s Disease Anti-inflammatory Prevention Trial (ADAPT) enrolled old cognitively unimpaired individuals and evaluated the effects of naproxen and celecoxib on cognitive function [79]. Although the participants were family members of AD patients, we did not know if they had AD, and cognitive impairment may not have started in them; hence, microglia and astrocytes may have been more neuroprotective than pro-inflammatory. The COX-inhibiting anti-inflammatory drugs may reduce microglial activation [82]. Furthermore, 3 years of follow-up may not be long enough for revealing a difference, as cognitive decline may not start in participants within 3 years. Minocycline, which can cross the BBB and inhibit pro-inflammatory microglia [83], showed beneficial effects on memory impairment caused by Aβ and reversed the increase in various inflammatory cytokines in an animal model of AD [84]. Recently, the Minocycline in Alzheimer’s Disease Efficacy (MADE) trial compared two different doses of minocycline and placebo in mild AD patients over 2 years, but failed to demonstrate a beneficial effect [85]. The complexity of the relationship between microglial activation and neurodegeneration and the minimal treatment effects of minocycline were considered as reasons for the negative result [86]. CHF 5074 (CSP-1103) is a modulator of microglia that polarizes microglia from the neurotoxic to the neuroprotective phenotype [38]. A CHF 5074 trial assessing mild cognitive impairment following AD is currently in phase II (NCT01421056). Cromolyn, which is used in patients with asthma, has been found to induce neuroprotective microglial activation, promote Aβ42 uptake in microglia, and reduce the aggregation-prone Aβ levels [39]. The cromolyn is currently under a phase III trial (NCT02547818) as a therapeutic for early stages of AD.

### PD

PD is the most frequent movement disorder and the second most frequent neurodegenerative disease after AD [6]. The accumulation of Lewy bodies, intracellular inclusions that contain α-synuclein, and dopaminergic neuronal death in the substantia nigra pars compacta and other brain regions are neuropathological hallmarks of PD [6, 87]. In addition, the activation of glial cells, including microglia and astrocytes, also contributes to the pathogenesis of PD. Several proteins that are encoded by genes associated with familial forms of PD, including α-synuclein (*PARK1* and *PARK4*), parkin (*PARK2*), DJ-1 (*PARK7*), and ATPase 13A2 (ATP13A2 gene), are involved in the regulation of microglial and astrocyte activation [6, 41].

The incremental activation of microglial cells (MHC-II-, ICAM-1-, and LFA01-positive cells) is observed in the substantia nigra of PD patients [88]. Furthermore, the degree of microglial activation is correlated with the dopaminergic terminal loss in early PD [89]. The activated microglia that surround dopaminergic neurons are generally pro-inflammatory [28]. The aggregated α-synuclein is released from dying dopaminergic neurons, and it activates microglia into the pro-inflammatory phenotype [90]. Over-expression of α-synuclein drives microglia into a reactive pro-inflammatory phenotype, and TNF-α, NO, and IL-1β derived from the pro-inflammatory microglia can modulate the neuroinflammatory process in PD [91, 92]. 1-Methyl-4-phenyl-1,2,3,6-tetrahydropyridine is known to cause dopaminergic neuron injury via mitochondrial dysfunction and by indirectly activating microglia [28, 93]. Lipopolysaccharide, a ligand of TLRs, can also cause dopaminergic neuronal death by activating the pro-inflammatory phenotype of microglia [28]. Jmjd3 has been reported to be essential for the expression of the M2 microglial phenotype [35]. Suppression of Jmjd3 attenuates the neuroprotective polarization and over-activates the pro-inflammatory microglial response with the exacerbation of dopaminergic neuronal cell death in a PD mouse model [35]. However, the role of the neuroprotective microglial phenotype is still unclear.

Reactive astrocytes have been detected in the substantia nigra pars compacta of PD patients [94]. Astrocyte dysfunction plays a role in dopaminergic neurodegeneration. Various genes are involved in the development of PD and astrocyte biology [95], including *PARK7* (encoding DJ-1), *SNCA* (encoding α-synuclein), *PARK2* (encoding Parkin), *PLA2G6* (encoding Ca^2+^-independent phospholipase A_2_), *ATP13A2* (encoding lysosomal type 5 ATPase, ATP13A2), *LRRK2* (encoding leucine-rich repeat kinase 2, LRRK2), *GBA* (encoding β-glucocerebrosidase, GCase), and *PINK1* (encoding PTEN-induced putative kinase 1, PINK1) genes [95]. The DJ-1 protein regulates astrocyte activation through the IFN-γ and TLR4 signaling [41, 96]. Maintaining the ATP13A2 level could prevent the activation of the NLPR3 inflammasome [41, 95].

Various anti-inflammatory treatments such as dexamethasone, ibuprofen, amantadine, minocycline, pituitary adenylate cyclase-activating peptide, vasoactive intestinal peptide, IL-10, and TGF-β have shown preventive effects on dopaminergic cell death in animal models [97–103]. However, the effects of anti-inflammatory drugs in PD patients are contradictory. One meta-analysis concluded that the nonsteroidal anti-inflammatory drugs (NSAIDs) may not modify the risk of PD, with only ibuprofen seeming to have a modest protective effect [104]. Another meta-analysis concluded that NSAIDs, except aspirin, may have a protective effect on the risk of PD [105]. Minocycline, which showed a neuroprotective effect in several in vivo and in vitro studies, was unsuccessful in altering the course of early PD over 12 and 18 months in a randomized clinical trial [106–108]. NLY01, a glucagon-like peptide-1 receptor agonist, was protective against dopaminergic neuronal loss and abnormal behavioral function in a sporadic PD mouse model [109]. This neuroprotective effect was attributed to the inhibition of the conversion of astrocytes to the neurotoxic phenotype, which was mediated by microglia [109].

### ALS

ALS, also called Lou Gehrig’s disease, is an adult-onset progressive neurodegenerative disease in which motor neurons are selectively affected [110]. The etiology of most ALS patients remains unidentified. Only less than 10% of cases are due to mutations of specific genes, including superoxidase dismutase 1 (*SOD1*), *C9orf72*, *TDP43*, and *FUS* [18]. Neuroinflammation is a pathological mechanism common to ALS patients with and without genetic mutations, which is characterized by the infiltration of activated microglia and astrocytes. The activated microglia and astrocytes that produce pro-inflammatory cytokines are upregulated in post-mortem tissues of ALS patients [41, 111, 112]. A PET study has demonstrated increases in activated microglia [^11^C-(R)PK11195 PET] and astrocytes (^11^C-DED PET) in living ALS patients [113, 114]. In addition, the CSF sTREM2 level is significantly higher in sporadic ALS patients with varying disease severity than controls [115]. In particular, the CSF sTREM2 level is highest in the early-stage ALS, and in late stage, higher levels of CSF sTREM2 are associated with slower disease progression [115]. Prolonged high levels of CSF sTREM2 may be indicative of a neuroprotective phenotype.

Toxicity caused by mutant *SOD1*, the most common form of inherited ALS, is mediated by direct damage that is incurred within the motor neurons, microglia, and astrocytes [110]. The activated pro-inflammatory microglia and astrocytes produce toxic factors that cause the initial damage and disease progression. The G930A-*SOD1* transgenic mouse model has demonstrated the ability of microglia to switch from the neuroprotective to the pro-inflammatory phenotype from the onset of the pathology [28, 116]. The *SOD1*-mutant microglia isolated from mice with early-stage ALS express higher levels of M2 microglia phenotype markers and lower levels of pro-inflammatory microglia markers, compared with the *SOD1*-mutant microglia isolated from mice with end-stage ALS [117]. Altogether, as ALS progresses, the function of neuroprotective microglia may decrease and the proportion of pro-inflammatory phenotypes may increase. The C3-expressing pro-inflammatory astrocytes and astrocytic NLRP3 inflammasomes have been found to be upregulated in post-mortem ALS patients [41, 46, 118]. The activation of astrocytes in ALS decreases their protective effects and increases their detrimental effects [119]. Astrocytes with *SOD1* mutations have been reported to release soluble factors toxic to motor neurons [120]. IL-1α, TNF-α, and C1q released from microglia drive astrocytes to the neurotoxic phenotype, while reducing reactive astrocytes by inhibiting these factors attenuates the disease progression in the G93A-*SOD1* mouse model [121]. However, little is known about the neuroprotective phenotype of astrocytes in the pathogenesis of ALS.

The ablation of NOX3 or NF-kB improved motor neuron survival in the G930A-*SOD1* transgenic mouse model [28, 122]. In addition, the administration of minocycline in G930A-*SOD1* transgenic mice selectively attenuated the expression of markers for the pro-inflammatory microglia, inhibited the upregulation of NF-κB in the primary culture of microglia, and delayed the pathogenesis [28, 123]. Cromolyn, which induced the activation of neuroprotective microglia in the AD mouse model, demonstrated a neuroprotective effect in the G93A-*SOD1* transgenic mouse model by delaying the disease onset and reducing the motor impairment [124].

Recently, masitinib, an oral tyrosine kinase inhibitor, has shown beneficial effects in ALS patients over 48 weeks [125]. Masitinib reduces the microgliosis and the emergence of aberrant glial cells in the G93A-*SOD1* transgenic mouse model [126]. Regulatory T-lymphocytes (Tregs) can augment IL-4 expression, induce the M2-phenotype microglia, and delay the progression of the disease [127]. Upregulation of Tregs can be achieved using dimethyl fumarate (Tecfidera), and a phase II trial of Tecfidera is being conducted in patients with sporadic ALS [128]. In addition, the infusion of tocilizumab in ALS patients reduced neuroinflammation [129], and it is now in a phase II clinical trial in ALS patients (NCT02469896), results of which are expected to be announced soon.

## Conclusion

We reviewed the roles of neuroinflammation in neurodegenerative diseases, focusing on microglia and astrocytes. In addition, clinical or experimental studies on treatments associated with neuroinflammation in neurodegenerative diseases were discussed. A balance between pro-inflammatory and neuroprotective glial cells may be critical in the progression of neurodegenerative diseases. Moreover, it has been reported that the activated microglia and reactive astrocytes influence each other. Due to the complexity of microglia and astrocyte phenotypes and the various types of drugs, the stages of neurodegenerative diseases (more pro-inflammatory than neuroprotective) and the conditions of patients (confirmed pathology of disease and likely to progress within few years) may be crucial for demonstrating the benefits of anti-inflammatory treatments in clinical trials. The functions of microglia and astrocytes at specific stages of specific diseases in specific patients need to be identified. The next step for trials is to determine a standard method for evaluating each phenotype of microglia and astrocytes to standardize further evaluation.

## Data Availability

Not applicable.

## Abbreviations

^11^C-DED: ^11^C-deuterium-L-deprenyl
Aβ: Amyloid-beta
Act 1: Activator 1
AD: Alzheimer’s disease
ALS: Amyotrophic lateral sclerosis
Arg1: Arginase 1
BBB: Blood-brain barrier
BDNF: Brain-derived neurotrophic factor
Chi3l3: Chitinase-3-Like-3
CCL: C-C motif chemokine ligand
CSF: Cerebrospinal fluid
CXCL: C-X-C motif chemokine ligand
DAMPs: damage-associated molecular patterns
EAE: Experimental autoimmune encephalomyelitis
ER: Oestrogen receptor
Fzd1: Frizzled class receptor 1
GFAP: Glial fibrillary acidic protein
GM-CSF: Granulocyte-macrophage colony-stimulating factor
IFN: Interferon
IGF-1: Insulin-like growth factor 1
IL: Interleukin
JAK/STAT: Janus kinase/signal transducers and activators of transcription
Jmjd3: Jumonji domain containing 3
LacCer: Lactosylceraide
LPS: Lipopolysaccharide
NFκB: Nuclear factor κB
NO: Nitric oxide
PAMP: Pathogen-associated molecular pattern
PD: Parkinson’s disease
PET: Positron emission tomography
SCI: Spinal cord injury
SOD1: Superoxidase dismutase 1
STAT3: Signal transducer and activator of transcription 3
sTREM2: Soluble triggering receptor expressed on myeloid cells 2
TGF: Transforming growth factor
TLR: Toll-like receptor
TNF: Tumor necrosis factor
TrkB: Tropomyosin receptor kinase B
Tregs: Regulatory T cells
